# Sago-Starch-Derived Sodium Starch Glycolate: An Effective Superdisintegrant to Enhance Formulation Performance

**DOI:** 10.3390/polym17091208

**Published:** 2025-04-28

**Authors:** Okta Nama Putra, Ida Musfiroh, Derina Paramitasari, Karjawan Pudjianto, Emmy Hainida Khairul Ikram, Chaidir Chaidir, Muchtaridi Muchtaridi

**Affiliations:** 1 Doctoral Program of Pharmacy, Department of Pharmaceutical Analysis and Medicinal Chemistry, Faculty of Pharmacy, Universitas Padjadjaran, Jatinangor 45363, Indonesia; okta22001@mail.unpad.ac.id (O.N.P.); ida.musfiroh@unpad.ac.id (I.M.); 2 Research Center for Agroindustry, Research Organization for Agriculture and Food, National Research and Innovation Agency (BRIN), Tangerang 15314, Indonesia; deri003@brin.go.id (D.P.); karj001@brin.go.id (K.P.); 3 Centre for Dietetics Studies, and Integrated Nutrition Science and Therapy Research Group (INSPIRE), Faculty of Health Sciences, Universiti Teknologi MARA Cawangan Selangor Kampus Puncak Alam, Bandar Puncak Alam 42300, Selangor, Malaysia; emmy4546@uitm.edu.my; 4 Research Center for Pharmaceutical Ingredient and Traditional Medicine, National Research and Innovation Agency, Tangerang 15314, Indonesia; chai001@brin.go.id; 5 Research Collaboration Centre for Radiopharmaceuticals Theranostic, National Research and Innovation Agency (BRIN), Jl. Soekarno KM-21, Jatinangor 45363, Indonesia

**Keywords:** response surface methodology (RSM), degree of substitution (DS), organic solvent slurry, mefenamic acid tablet formulation, disintegration time, sustainable agriculture

## Abstract

This study focused on optimizing sago-starch-derived sodium starch glycolate (SSG) as a superdisintegrant using a Response Surface Methodology (RSM). The aim was to enhance the formulation performance by achieving an optimal degree of substitution (DS) in the synthesis of SSG from sago starch and evaluating its performance in mefenamic acid tablet formulation. The SSG was synthesized using an organic solvent slurry method, which involves crosslinking starch with sodium trimetaphosphate (STMP) and substituting it with sodium monochloroacetate (SMCA). The reaction conditions, including the temperature, SMCA ratio, and reaction time, were optimized using the RSM. The optimal conditions were identified as a temperature range of 45–55 °C, an SMCA ratio of 0.75–1.5, and a reaction time of 120–240 min. The maximum predicted DS value was 0.24, with a validated DS value of 0.246 ± 0.021. The SSG-containing mefenamic acid formulation met USP standards and showed a superior disintegration time compared to the existing SSG. The optimized SSG derived from sago starch can be effectively used as a superdisintegrant in pharmaceutical formulations, offering a sustainable and economically viable alternative source of SSG. This contributes to the development of more effective drug delivery systems and promotes sustainable agriculture in Indonesia.

## 1. Introduction

Drug delivery systems encompass a wide array of technologies and approaches that enable the precise delivery of medications to targeted sites within the body. These systems utilize diverse technologies and strategies to guarantee the precise timing, accurate dosage, and appropriate administration site of the drug. Drug delivery involves administering medication to patients in a manner that enhances the drug concentration in particular body regions, aiming to prolong, localize, and target drug action within diseased tissue while ensuring safe interactions [1]. Active pharmaceutical ingredients (APIs) are the key elements of drugs that produce the desired therapeutic outcomes; however, several APIs have poor delivery due to their physicochemical properties, such as low solubility and stability or rapid metabolism. These drawbacks could lead to several negative impacts during drug formulation and the therapeutic process. Inadequate physicochemical characteristics of APIs may impede the effectiveness of treatment, influencing the development of formulations and subsequent processes after formulation, which could result in negative effects and toxicity [2]. Therefore, excipients are used to overcome these drawbacks, modulating the bioavailability and solubility as well as improving the stability of APIs.

Excipients are essential for drug delivery as they perform a variety of functions in dosage forms. There are different functional classifications for excipients based on their intended use, including binders, diluents, disintegrants, lubricants, wetting agents, solvents, fillers, emulsifiers, absorption enhancers, sustained release matrices, preservatives, sweeteners, and stabilizing, coloring, or flavoring agents. Disintegrants, for example, play a crucial role in facilitating the swift disintegration of tablets or capsules after consumption, ensuring the effective liberation and assimilation of the active pharmaceutical ingredient. Disintegrant agents are added to drug formulations to aid in the dispersal of the tablet or capsule matrix into smaller pieces, leading to a greater surface area that enhances the speed of drug dissolution [3]. Moreover, superdisintegrants are more effective than disintegrants and at low dosages could provide the more rapid disintegration and dissolution of the tablet or capsule. One example of a superdisintegrant is sodium starch glycolate (SSG), a modified starch with the form of the sodium salt of a carboxymethyl ether of starch that can be produced from various kinds of starch, including potato, rice, wheat, or maize [4]. The carboxymethylation reaction, which introduces carboxymethyl groups into the starch structure, plays a fundamental role in enhancing the performance of SSG as a superdisintegrant. This chemical modification increases the material’s hydrophilicity and swelling capacity, enabling tablets to disintegrate more rapidly in aqueous environments. These properties are essential for ensuring the quick release and absorption of the active pharmaceutical ingredient.

Furthermore, the degree of substitution (DS)—which indicates the number of hydroxyl groups in the starch molecule that are replaced by carboxymethyl groups—must be carefully optimized. A DS that is too low may result in inadequate swelling and disintegration, while an excessively high DS can lead to high solubility and the reduced mechanical integrity of the excipient. Therefore, optimizing the DS is crucial to achieving an ideal balance that ensures effective tablet disintegration while maintaining formulation stability.

A potential alternative to local starch in Indonesia that could be utilized for producing SSG is sago starch. Sago starch, extracted from the pith of the sago palm (Metroxylon Sago), is a substance high in carbohydrate that is commonly used as a thickening agent and stabilizer in food industries. Indonesia has a remarkable abundance of sago starch, achieving 82.5–132 million tons of dry sago starch annually [5]. In addition, sago palm is a prime example of a sustainable plant because of its endurance in harsh environmental conditions and its ability to counteract the greenhouse effect [6]. Given the substantial availability of sago starch in Indonesia, there is a considerable opportunity for its utilization beyond food applications, for example, as a key superdisintegrant used in pharmaceutical formulations. Exploring the feasibility of deriving SSG from sago starch could unlock numerous benefits, such as increasing the added value of sago starch, creating potential new economic opportunities in pharmaceutical industries, and promoting the sustainable development of Indonesian agriculture.

The potential of sago starch utilization for SSG production remains largely unexplored, especially in locales where sago palms are prevalent, such as Indonesia. In pursuit of obtaining high-quality SSG products through an effective process design, an innovative statistical and mathematical design method known as the Response Surface Methodology (RSM) was developed. The RSM is a statistical approach employed for process optimization through the systematic adjustment of input variables in order to attain the desired output responses. This method is especially valuable for investigating intricate relationships and interdependencies among various factors to enhance the quality of a product.

This study aimed to optimize the production of sodium starch glycolate (SSG) from sago starch using the Response Surface Methodology (RSM) and to achieve the desired SSG specifications with accuracy and reliability [7]. The process parameters investigated included the reaction temperature, the ratio of sodium monochloroacetate (SMCA), and the reaction time. The validated SSG product was characterized by the amylose content, functional properties, and its performance in the formulation and evaluation of mefenamic acid tablets.

The validated SSG product was characterized by its amylose content, functional properties, and its performance in the formulation and evaluation of mefenamic acid tablets. Traditionally, SSG is derived from corn or potato starch through a two-step process, where starch is modified with glycols, like propylene glycol or glycolic acid, and then dissolved using sodium. While this conventional method is widely used, it presents several limitations, including dependency on natural starch sources like corn and potatoes. Fluctuations in the availability of these raw materials can affect SSG production. Additionally, the complex modification process, involving multiple steps and stringent quality control measures, increases production costs.

This research, however, introduces an innovative approach for producing superdisintegrants using SSG derived from a promising local resource—sago starch. The method employed, the organic solvent slurry method, offers several advantages, including improved efficiency and enhanced product quality. Furthermore, this method has the potential to reduce waste, lower the environmental impact, and diversify raw material sources by incorporating the organic slurry method with STMP and SMCA. Ultimately, improving the efficiency and quality of SSG production not only enhances manufacturing processes but also contributes to the sustainability of the pharmaceutical industry.

## 2. Materials and Methods

### 2.1. Materials

Native sago starch was sourced from PT. Bangka Asindo Agri. All other chemicals used in this study—including sodium trimetaphosphate (STMP), sodium monochloroacetate (SMCA), isopropyl alcohol, ethanol, sodium sulfate, sodium hydroxide, hydrochloric acid, and glacial acetic acid—were of analytical grade and purchased from Merck (Rahway, NJ, USA).

### 2.2. Experimental Design

The optimization of the molar substitution (MS) in the synthesis of sodium starch glycolate (SSG) was designed using the Response Surface Methodology (RSM) with a Central Composite Design (CCD) framework. This approach aimed to model the response surface and perform a statistical evaluation of the process in Table 1. Three independent variables were selected: the reaction temperature (A1), the ratio of sodium monochloroacetate (SMCA) (A2), and the reaction time (A3). The selected ranges for each variable were 45–55 °C for A1, 0.75–1.5 for A2, and 120–240 min for A3. Design Expert version 13 (Stat-Ease Inc., Minneapolis, MN, USA) was used to generate 20 experimental runs, consisting of 14 factorial (non-center) points and 6 center points. Each experiment was performed in triplicate, and the results are presented as mean values accompanied by standard deviations.

The relationship between the response (MS) and the independent variables was described using a second-order polynomial model. This equation, derived from the RSM analysis, represents how the three variables collectively influence the degree of molar substitution, as shown in Equation (2):

(1) $$R=\beta_{0}\pm \Sigma_{i=1}^{k}\;\beta_{i}{\;*\;A}_{i}\pm \Sigma_{i=1}^{k-1}\Sigma_{j=i+1}^{k}\;\beta_{ij}\;*\;A_{i}\;*\;A_{j}\pm \;\Sigma_{i=1}^{k}\beta_{ii}\;*\;A_{i}^{2}$$

(2) $$\;R=\beta_{0}+\beta_{1}\;*\;A_{1}+\beta_{2}\;*\;A_{2}+\beta_{3}\;*\;A_{3}+\beta_{12}\;*\;A_{1}\;*\;A_{2}+\beta_{13}\;*\;A_{1}\;*\;A_{3}+\beta_{23}\;*\;A_{2}\;*\;\;\;\;\;\;\;\;\;\;\;\;\;\;\;\;\;\;\;\;\;\;\;\;\;\;\;A_{3}+\beta_{11}{\;*\;A}_{1}^{2}+\beta_{22}{\;*\;A}_{2}^{2}+\beta_{33}\;*\;A_{3}^{2}$$

In the model equation, *R* denotes the response variable. The term *β*_0_ represents the intercept or constant, *β_i_* corresponds to the coefficients of the linear effects, *β_ii_* refers to the coefficients of the quadratic effects, and *β_ij_* indicates the coefficients for the interaction effects between variables. The symbols *A_i_* and *A_j_* are the coded forms of the independent variables, while K signifies the total number of variables included in the model.

### 2.3. Crosslinking of Sago Starch

To prepare a 40% *w*/*w* starch suspension, 100 g of native sago starch (NSS) was mixed with 10% *w*/*w* sodium sulfate (Na_2_SO_4_) and 12% *w*/*w* sodium trimetaphosphate (STMP) in a reaction flask. The mixture was stirred thoroughly until a uniform suspension was formed. Thereafter, 1 M sodium hydroxide (NaOH) was added dropwise to adjust the pH to between 11.5 and 13. The solution was continuously stirred in a closed system at the ambient temperature for 30 min.

Following this step, the suspension was transferred to a water bath, heated to 45 °C, and shaken at 150 rpm for a duration of 180 min. The reaction was terminated by gradually neutralizing the mixture with 0.5 M hydrochloric acid (HCl) until the pH reached 6.5–7. The final product was recovered through filtration and subjected to repeated washing using ethanol and distilled water. The resulting solid was then dried in an oven at 50 °C overnight until its moisture content was reduced to below 12% *w*/*w*.

### 2.4. Carboxymethyl Substitution of Sago Starch

Starch crosslinking is achieved through the substitution of chloroacetic acid or sodium monochloroacetate in an alkaline alcohol suspension, following the principles of Williamson’s ether synthesis.

The carboxymethylation process was initiated by preparing a suspension containing 100 g of crosslinked sago starch mixed with isopropyl alcohol, stirred until a uniform consistency was achieved. The pH of the mixture was then adjusted to 11.5–13 using 1 M sodium hydroxide (NaOH), and the slurry was stirred continuously in a sealed flask at room temperature for 30 min to ensure complete homogenization.

Sodium monochloroacetate (SMCA) was subsequently added in accordance with the ratios specified in the experimental design Table 2. The reaction mixture was maintained at the appropriate temperature and reaction time as determined by the experimental parameters. After the alkaline slurry was prepared, SMCA was introduced gradually while the mixture was stirred constantly, then left to stand for 15 min at room temperature in a closed system.

The suspension was heated and agitated at 150 rpm in a water bath for the designated reaction duration outlined in Table 2. To stop the reaction, the mixture was neutralized with 0.5 M hydrochloric acid (HCl) until the pH reached between 6.5 and 7. The product was then filtered and washed thoroughly several times with ethanol and distilled water. The solid was dried in an oven at 50 °C overnight until its moisture content was reduced to no more than 12% *w*/*w*. At the end of this process, sodium starch glycolate was successfully obtained and isolated.

### 2.5. Amylose Content

The amylose contents in the NSS and the SSG were measured using the method reported by Mathew et al. [8]. To determine the amylose content, a starch suspension was prepared by dissolving 0.1 g of the starch sample in a mixture of 1 mL of 95% ethanol and 9 mL of 1 M sodium hydroxide (NaOH), ensuring complete homogenization. The resulting mixture was heated in a boiling water bath for 15 min, then allowed to cool to room temperature. After cooling, the solution was transferred to a 100 mL volumetric flask and diluted to the mark with distilled water.

A 5 mL aliquot of this solution was then pipetted into a separate 100 mL volumetric flask. To this, 1 mL of 1 M acetic acid and 2 mL of iodine solution were added. The mixture was diluted to volume with distilled water, mixed thoroughly, and left to stand for 20 min to allow for color development. The absorbance of the resulting solution was measured at 620 nm using a spectrophotometer. The amylose concentration was quantified by referencing a standard calibration curve prepared from known ratios of amylose and amylopectin.

### 2.6. Degree of Substitution of SSG Sago

Following the procedure described in reference, the degree of substitution (DS) was calculated [9]. A 50 mg sample was digested by adding 4 cm^3^ of concentrated nitric acid in a glass container placed on a heated plate. After digestion, the solution was diluted with deionized water to a final volume of 100 cm^3^. The sodium content was then analyzed using a flame atomic absorption spectrometer (AAS), employing an air–acetylene flame. The absorbance was measured at a wavelength of 589.0 nm, which corresponds to the sodium emission line. The degree of substitution was calculated based on the measured sodium content using the following procedure:

(3) $$\mathrm{DS}=\frac{162\%\;Na}{\left(2300-80\%\;Na\right)}$$

The %*Na* of the unmodified starch was previously determined and corrected with SSG derivatives [10]. The degree of substitution (DS) for sodium starch glycolate typically falls within the range of 0.23 to 0.32, which is considerably lower than the theoretical maximum value of 3. This maximum represents the complete substitution of all three hydroxyl groups on each anhydroglucose unit.

### 2.7. NaCl Measurement of SSG Sago

During the synthesis process of superdisintegrants, it is possible for by-product impurities to be generated. The synthesis of sodium starch glycolate (SSG) generates several by-products, including sodium chloride, sodium glycolate, and sodium citrate. According to official pharmacopeial standards, such as USP 32/NF 27 and PhEur 6.0, the allowable limits for these by-products are clearly defined. Specifically, the sodium chloride content must not exceed 7%, while the sodium glycolate concentration should remain below 2% [11].

The concentration of sodium chloride (NaCl) in the refined carboxymethyl starch was determined based on the procedure outlined in the *British Pharmacopoeia*. A suspension was prepared by dissolving 0.5 g of purified carboxymethyl starch in 100 mL of distilled water containing 1 mL of nitric acid. The suspension was then analyzed by potentiometric titration using a 0.1 M silver nitrate solution and a silver indicator electrode. In this method, 1 mL of 0.1 M silver nitrate is chemically equivalent to 5.844 milligrams of sodium chloride.

The NaCl content was calculated using the following formula:

(4) $$\mathrm{NaCl}\;(\%)=\frac{V1\;\frac{5844}{1000}}{w1}\times 100$$

Here, *V*_1_ (mL) refers to the volume of 0.1 M silver nitrate solution required to reach the endpoint of the titration, while *W*_1_ (g) denotes the weight of the carboxymethyl starch (CMS) sample used in the analysis [12].

### 2.8. Fourier Transform Infrared Spectroscopy (FTIR)

Fourier Transform Infrared (FTIR) Spectroscopy (Alpha II, Bruker, Karlsruhe, Germany) was employed to characterize the functional groups in both the NSS and the SSG samples. A powdered sample weighing between 0.1 and 0.5 g was carefully applied onto the diamond ATR crystal using a stainless-steel spatula or a comparable laboratory instrument. The sample was secured using a pressure anvil to ensure a firm contact with the crystal. The FTIR spectra were recorded across a wavenumber range of 400 to 4000 cm^−1^ to detect absorption bands corresponding to specific molecular vibrations within the sample [4].

### 2.9. Scanning Electron Microscopy Analysis

The surface morphology of the samples was analyzed using an Environmental Scanning Electron Microscope (E-SEM, Quattro S, Thermo Fisher Scientific, Waltham, MA, USA). The powdered samples were mounted onto a 12.5 mm diameter aluminum pin stub and then positioned on a designated holder within the instrument chamber. Imaging was performed at an accelerating voltage of 20 kV with a magnification of 1000×.

### 2.10. Differential Scanning Calorimetry Analysis (DSC)

The thermal properties of the NSS and SSG samples were evaluated using differential scanning calorimetry (DSC) with a Mettler Toledo MSC 3+ instrument (Greifensee Schweiz, Uster, Switzerland), as described by Ariyantoro et al. [13] with slight modifications. A 5 mg sample was placed into an aluminum DSC pan, and 5 µL of distilled water was added using a syringe to ensure precise measurement. The sample was then heated from 32 °C to 200 °C at a rate of 10 °C per minute under a nitrogen gas flow of 40 mL/min. An empty, sealed aluminum pan was used as the reference. The thermal parameters obtained from the DSC thermograms included the onset temperature (To), peak temperature (Tp), endset temperature (Te), and gelatinization enthalpy (ΔH), as calculated using the instrument software.

### 2.11. Pasting Properties

The gelatinization profile of the starch samples was analyzed using a Brabender Amylograph-E (Brabender GmbH & Co. KG, Duisburg, Germany). A suspension containing 6% *w*/*w* of starch solids was prepared by thoroughly mixing the sample with distilled water, then it was loaded into the mixing bowl of the instrument. The analysis was conducted following a programmed thermal cycle, which involved heating from 30 °C to 93 °C, holding at 93 °C for 20 min, cooling down to 50 °C, and then maintaining that temperature for an additional 20 min. Throughout the test, the mixing blade rotated at a constant speed of 75 rpm, while the heating rate was maintained at 1.5 °C per minute. A torque of 700 cm-g (equivalent to 1000 Brabender Units) was applied.

Key pasting parameters—including the pasting temperature, peak viscosity, final viscosity, setback viscosity, and breakdown viscosity—were automatically recorded and quantified by the Brabender system software in Brabender Units (BU).

### 2.12. Water Solubility and Swelling Power

The swelling power and solubility of NSS and SSG were assessed using a modified version of the method outlined by Fouladi and Nafchi [14]. A 0.1 g sample (dry basis) was combined with 10 mL of distilled water in a 50 mL centrifuge tube. The mixture was thoroughly homogenized and then incubated in a shaking water bath at 70 °C for 30 min. Following the heating step, the suspension was allowed to cool to the ambient temperature and subsequently centrifuged at 2500 rpm for 60 min using a Hitachi 05P-21 centrifuge (Japan).

(5) $$SP=\frac{W_{1}}{W_{0}}\;\;$$

(6) $$S=\frac{W_{2}}{W_{0}}\times 100$$

In these equations, *SP* represents the swelling power expressed in grams per gram (g/g), and S denotes the solubility percentage. *W*_1_ is the weight of the sediment (in grams), *W*_2_ refers to the weight of the dried supernatant (in grams), and W_0_ indicates the initial weight of the sample (in grams).

### 2.13. X-Ray Diffraction Analysis—Crystallinity

The crystallinity and diffraction patterns of the materials were analyzed using X-ray diffraction (XRD) with an X’Pert3 powder diffractometer (Malvern Panalytical, Almelo, The Netherlands). Measurements were conducted over a 2θ range of 10–40°, using a scanning rate of 20 rpm, with an operating current of 20 mA and a voltage of 40 kV.

### 2.14. Mefenamic Acid Tablet Formulation

The formulations of the fast-disintegrating tablets (FDTs) were prepared using the direct compression method. Granulated mannitol was used as the diluent, sodium starch glycolate served as the superdisintegrant, and Avicel^®^ PH-102 was employed as the filler. Additional excipients included talc and magnesium stearate. For the different FDT formulations—using either SSG derived from sago starch, SSG derived from potato starch from Gujarat, or no SSG—all the ingredients (the active pharmaceutical ingredient, SSG, mannitol, and microcrystalline cellulose) were blended using a V-mixer. The talc and magnesium stearate were added afterward. The resulting powder blend was evaluated for flow properties and compressibility, then compressed into tablets with a target weight of 700 mg.

### 2.15. Evaluation of Tablets

All the tablet formulations were assessed for their organoleptic properties, hardness, and disintegration time. The organoleptic evaluation included the visual inspection of the tablets’ size, shape, surface texture, color, and the presence of any physical imperfections. For hardness testing, each tablet was positioned appropriately in a hardness tester. Force was gradually applied until the tablet fractured, and the breaking force was recorded in kilograms. Disintegration testing was performed using ten tablet samples, each placed into a cylindrical tube equipped with a disk. The test was conducted in warm water maintained at 37 ± 2 °C as the disintegration medium. The apparatus was activated, and the time required for complete disintegration—defined as the absence of any intact or translucent core—was recorded. The disintegration test was conducted using distilled water at 37 ± 2 °C as the testing medium, following the general disintegration procedures outlined in the *United States Pharmacopeia* (USP). Neither simulated gastric fluid (SGF) nor simulated intestinal fluid (SIF) were used in this preliminary study.

### 2.16. Statistical Analysis

All the numerical data from the functional characterization of NSS and SSG were obtained from triplicate measurements and are presented as mean values ± standard deviations, unless otherwise specified. The validated HPPS method was employed for the analysis of the functional properties. Statistical significance among the sample groups was determined using a one-way Analysis of Variance (ANOVA), followed by Tukey’s post-hoc test at a significance level of *p* < 0.05. All the statistical analyses were performed using Minitab software version 12.4.1 (State College, PA, USA).

## 3. Results

### 3.1. Statistical and Model Fitting of SSG Derived from Sago Starch

Table 1 summarizes the statistical analysis of the molar substitution (MS) for sodium starch glycolate derived from sago starch. The optimal polynomial model was identified based on several selection criteria, including the use of a higher-order model, low standard deviation, a non-significant lack-of-fit (*p* > 0.05), a high coefficient of determination (R^2^), a low predicted residual error sum of squares (PRESS), and a highly significant model *p*-value (*p* < 0.0001).

The quadratic model was selected over the cubic model as it demonstrated a highly significant *p*-value (less than 0.0001) and was free from aliasing. The equation representing the quadratic model in terms of actual variables is presented below.

$$DS=-1.91984+0.091084\times T-0.480533\times S-0.002686\times t\;\;\;\;\;\;\;\;\;+0.008432*T*S+0.000015*T*+0.000653*S*t\;\;\;\;\;\;\;\;\;-0.000924*T^{2}+0.018007*S^{2}+4.29672E-06*t^{2}$$

where *T* is the reaction temperature, *S* is the SMCA ratio, and *t* is the reaction time in minutes.

The response (DS) was modeled using a second-order polynomial equation derived from the Response Surface Methodology (RSM), consisting of linear, interaction, and quadratic (squared) terms to evaluate the effects of the temperature (A), SMCA ratio (B), and reaction time (C). The effect of each independent variable on the response was interpreted based on the direction (sign) and size (magnitude) of its regression coefficient. A positive coefficient signifies that an increase in the variable leads to an increase in the response, whereas a negative coefficient indicates that a higher value of the variable results in a reduction in the response. In this study, the linear terms S and t, along with the quadratic term T^2^, demonstrated a negative influence on the degree of substitution (DS). Conversely, for variables such as T, the interactions T × S, T × t, and S × t exhibited a positive contribution. Similarly, the quadratic terms S^2^ and t^2^ were also found to enhance the DS. The highest DS value observed was 0.356 in Experiment 8, while the lowest was 0.057 in Experiment 9. A summary of the model’s predicted DS values is provided in Table 2.

### 3.2. Analysis of Variance (ANOVA)

The statistical significance of the response model was evaluated using an ANOVA, as generated by the Design Expert software and summarized in Table 3. The model was deemed significant due to its high F-value and a *p*-value of less than 0.05 [15]. The F-value, which represents the ratio of the variance between groups to the variance within groups, was calculated to be 57.09. This high value suggests the model is statistically relevant, with only a 0.01% probability that such a result could have occurred by chance.

Further analysis showed that the terms A, B, C, AB, BC, A^2^, and C^2^ had significant effects on the response variable, as indicated by *p*-values below 0.05. Conversely, the terms AC and B^2^ were not statistically significant, with *p*-values exceeding 0.100.

The model’s goodness-of-fit was assessed using several metrics, including the coefficient of determination (R^2^), adjusted R^2^, predicted R^2^, adequate precision, and coefficient of variation (CV). The R^2^ value of 0.9809 indicates that 98.09% of the variability in the response could be explained by the model. Since R^2^ can artificially increase with the addition of variables, the adjusted R^2^ (0.9637) provides a more accurate assessment of model performance by penalizing irrelevant terms. The predicted R^2^ value of 0.8864 is within an acceptable range of the adjusted R^2^, suggesting the model has a good predictive capability.

The adequate precision, which measures the signal-to-noise ratio, was 33.5819—well above the acceptable threshold of 4—indicating a strong and reliable signal. The model’s coefficient of variation (CV) was 6.22%, which falls below the commonly accepted 10% limit, confirming the model’s precision and reproducibility [16]. Additionally, the lack-of-fit (LOF) test yielded a *p*-value of 0.2140, indicating that the LOF is not significant and the model fits the experimental data well.

### 3.3. Model Adequacy Evaluation

**Figure 1:**
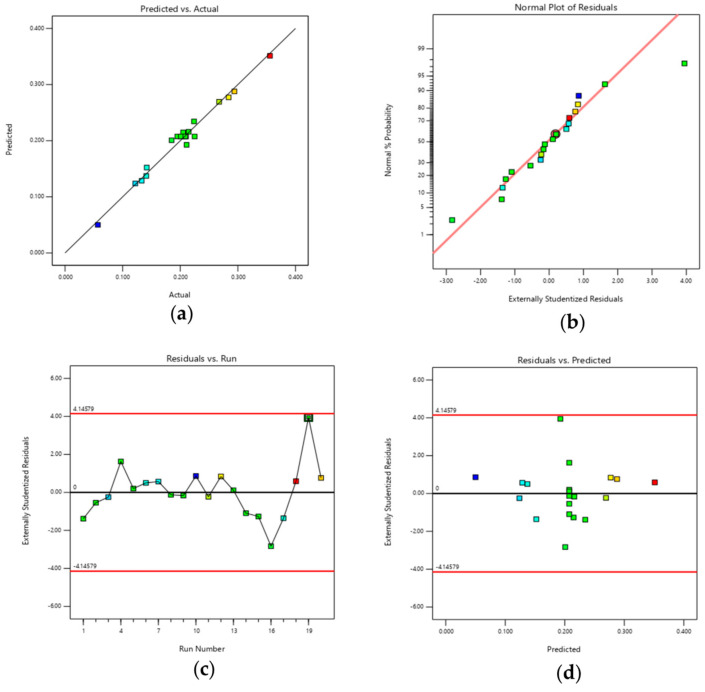
Diagnostic plots derived from the Central Composite Design (CCD) for the optimization of SSG processing conditions, which include (**a**) predicted versus actual values, (**b**) a normal probability plot of residuals, (**c**) residuals plotted against the run number, and (**d**) residuals versus predicted values.

The predicted DS values generated by the response model showed strong agreement with the experimental data, as illustrated in Figure 1. The model’s validity was further assessed using several diagnostic plots, including predicted vs. actual values, a normal probability plot, and externally studentized residuals, shown in Figure 1a–d.

In Figure 1a, the predicted values align closely with the regression line, indicating strong consistency between the model and the experimental results. The normal probability plot (Figure 1b) shows that the residuals are approximately normally distributed, as they follow the diagonal line, suggesting that the model is statistically sound.

Figure 1c displays the residuals from all the experimental runs, which appear randomly dispersed within a narrow and consistent range (+4.14579 to −4.14579), indicating the absence of any systematic pattern or bias. This supports the assumption of independence and constant variance across the dataset.

Furthermore, Figure 1d illustrates the externally studentized residuals plotted against the predicted response values. The residuals are randomly scattered within a consistent range, reinforcing the homogeneity of variance and confirming the model’s adequacy and reliability for prediction.

### 3.4. Effects of Three Parametric Processes on DS

The statistical significance of the quadratic model representing DS suggests a robust relationship between the parametric processes and the DS response. The linear effects of reaction temperature, SMCA/starch ratio, and reaction time, alongside the quadratic impacts of the reaction temperature and the reaction time, as well as the interactive effects of the reaction temperature vs. the SMCA/starch ratio and the SMCA/starch ratio vs. the reaction time, all significantly contribute to the DS in terms of carboxymethyl groups. The DS is a critical material attribute for sodium starch glycolate as an excipient (disintegrant) [17], with reported values ranging between 0.23 and 0.32 [18]. The degree of substitution correlates with the total acidic (acid form) and basic (sodium salt) components of superdisintegrants [17].

#### 3.4.1. Effect of Reaction Temperature

**Figure 2:**
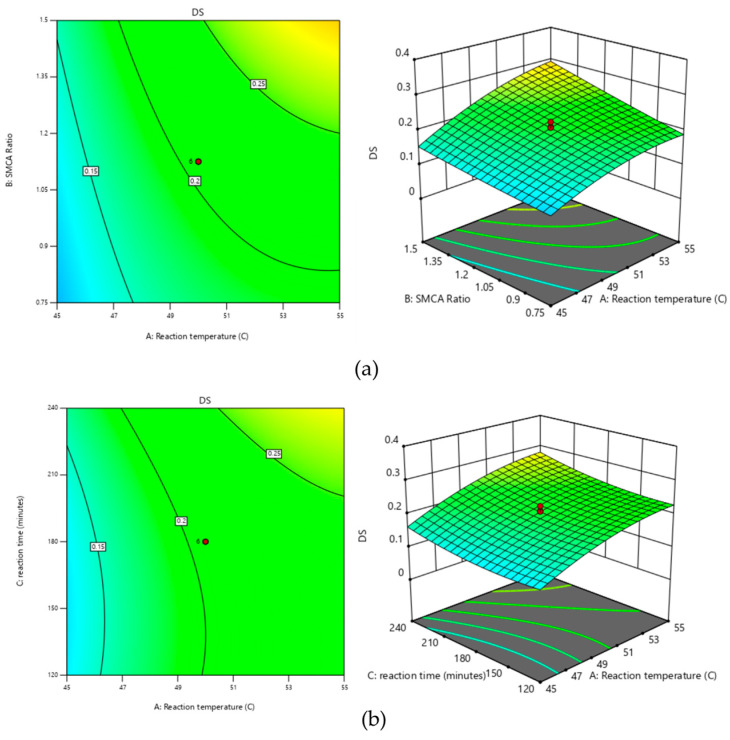

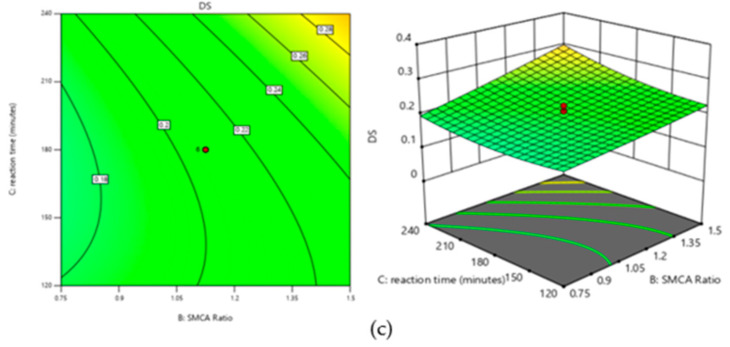
Response surface curves for effects of (**a**) reaction temperature (°C) and SMCA ratio, (**b**) reaction temperature (°C) and reaction time (minutes), and (**c**) reaction time (minutes) and SMCA ratio due to DS value.

The reaction temperature had significant linear and square effects on the DS, as well as the interaction impacts of the reaction temperature and the SMCA/starch ratio. The higher the reaction temperature, the greater the DS (Figure 2a,b). Increasing the temperature of a chemical reaction generally accelerates the reaction rate. As the reactant molecules gain heat, they move more rapidly, leading to more frequent collisions. More importantly, these collisions occur with greater energy, making it more likely for the reactants to overcome the activation energy barrier and form products. Thus, a rise in temperature not only increases the number of collisions but also enhances the proportion of collisions that are effective, ultimately resulting in a faster reaction rate [19]. Increasing the reaction rate would increase the DS. The chemical reaction is an endothermic reaction that requires heat to react, corresponding to the Arrhenius equation [20]:

(7) $$k=Ae^{\frac{-EA}{RT}}$$

k = rate constant; A = pre-exponential factor; EA = activation energy (J·mol^−1^); R = gas constant (J·mol^−1^·K^−1^); T = temperature (K).

The reaction temperature needed to be carefully controlled to prevent starch gelatinization, as gelatinization in the solvent could hinder subsequent recovery and drying processes [12]. The optimal temperature for the carboxymethylation of sago starch was determined to be 55 °C.

#### 3.4.2. Effect of SMCA/Starch Ratio

The linear effect of the SMCA/starch ratio on the degree of substitution (DS) is significant, along with the interactive effects between the reaction temperature and the SMCA/starch ratio, as well as between the SMCA/starch ratio and reaction time. A higher SMCA/starch ratio increases the SMCA concentration, leading to a higher DS (Figure 2a,c). These results were similar to previous studies reported by Hebeish and Khalil [21] and Bhattacharyya et al. [22]. In general, increasing the concentration of reactants leads to a higher reaction rate. This is because a greater number of particles within a given volume results in more frequent collisions between them. Since the reaction rate depends on the frequency of effective collisions among reactant molecules, higher concentrations typically accelerate the reaction [19]. The higher the reaction rate, the greater the DS.

#### 3.4.3. Effect of Reaction Time

The reaction time exhibited significant linear and quadratic effects on the DS, alongside interactive effects with the SMCA/starch ratio. Prolonged reaction times can increase the DS (Figure 2b,c) by allowing more time for the SMCA and the starch to react, thereby substituting more carboxymethyl groups onto the hydroxyl groups of starch.

### 3.5. Model Optimization and Validation of Response Surface Methodology for Degree of Substitution

The optimal degree of substitution (DS) for sodium starch glycolate (SSG) was identified using the Response Surface Methodology (RSM) model developed in Design Expert version 13. The model optimization targeted a DS value of 0.24, with the three processing variables confined to the predefined ranges specified in the experimental design. To validate the model, experiments were performed in triplicate under these optimized conditions, and the resulting DS values were averaged and expressed with standard deviations to assess the agreement between the experimental outcomes and the predicted values.

The process conditions that produced the highest predicted DS value of 0.24—accompanied by a desirability score of 1.0—included a reaction temperature of 55 °C, an SMCA ratio of 1.185, and a reaction time of 125 min. To verify the reliability of the model, experimental validation was performed using these optimized parameters. The resulting DS value obtained experimentally was 0.246 ± 0.021; to validate the accuracy of the predicted DS value (0.24), the experimental trials were conducted in triplicate (n = 3) under the optimized conditions obtained from the RSM model, specifically at a reaction temperature of 55 °C, an SMCA ratio of 1.185, and a reaction time of 125 min. The resulting experimental DS was 0.246 ± 0.021, presented as the mean ± standard deviation. This variation reflects minor experimental errors arising from instrumental sensitivity, reagent handling, and process control variability (e.g., temperature fluctuation and timing accuracy). All the validation experiments were carried out using identical batches of materials and standardized laboratory procedures to ensure their reproducibility and minimize any bias. These findings confirm the robustness and predictive reliability of the developed model, indicating that the model had a high precision and is eligible for use. These findings indicate that the model demonstrated high precision and was suitable for the application. In this study, the synthesis of sodium starch glycolate from sago starch was optimized to achieve a DS value of 2.4, using a reduced amount of sodium monochloroacetate (SMCA) and a shorter reaction time when compared to a previous study on sodium carboxymethyl starch derived from Ipomoea batatas conducted by M. Achor [10]. Specifically, the previous study required an SMCA ratio of 1:8 for sago crosslinking and 6 h of reaction time, yet the DS value obtained was only 0.52. A related study focusing on the optimization of carboxymethylation conditions for sago starch was also successfully conducted. The optimized parameters included a 20% aqueous NaOH concentration, a reaction time of 1 h, a reaction temperature of 55 °C, and a molar ratio of SMCA to anhydroglucose units (AGUs) of 1.5:1. Under these conditions, the process achieved a degree of substitution (DS) of 1.05 and a reaction efficiency of 85.9% [23].

## 4. Discussion

### 4.1. Functional Characterization of Sago Starch Glycolate

#### 4.1.1. Amylose Content, DS, and NaCl Measurement

The effects of carboxymethylation on the amylose content of native sago starch (NSS) and sodium starch glycolate (SSG) are summarized in Table 3, Table 4 and Table 5. The amylose content of NSS was measured at 20.7%, which is consistent with the 21.9% previously reported by Srichuwong et al. [24]. Sago starch is generally characterized by a high amylopectin content (70–80%) and a relatively low amylose content, typically of between 15–30% [25].

The modification processes—namely crosslinking followed by carboxymethylation—resulted in a substantial reduction in the amylose content. After crosslinking, the amylose level decreased to 6.10% and further declined to 3.71% following the second carboxymethylation step. These findings demonstrate that chemical modification significantly alters the structural composition of the starch. This significant reduction in the amylose content following carboxymethylation can be attributed to the cleavage and substitution of linear amylose chains, which disrupt the ordered crystalline structure and transform the starch into a more amorphous and hydrophilic form. Mechanistically, the decreased amylose content reduces the tendency for retrogradation and gel formation, thus enhancing water uptake and particle swelling when exposed to aqueous environments. These structural changes facilitate faster tablet disintegration by allowing rapid water penetration and the expansion of the starch granules. As a result, the modified SSG exhibits improved disintegration efficiency, a critical property for its function as a superdisintegrant in tablet formulations.

This phenomenon can be attributed to the incorporation of sodium monochloroacetate and sodium trimetaphosphate groups into the starch backbone during the crosslinking and carboxymethylation processes. These chemical modifications introduce sodium salt groups into the starch chains, which disrupt the original structure—particularly the linear configuration of amylose. As a result, carboxymethylated starch exhibits a more branched and heterogeneous molecular structure compared to native starch.

Carboxymethylation shortens amylose chains and alters their linearity, leading to significant changes in the physicochemical properties of the starch. These include increased branching, enhanced viscosity, improved water absorption, better thermal stability, and modified gelling behavior. Such characteristics make carboxymethylated starch highly suitable for various food and industrial applications, especially in formulations requiring rapid disintegration, such as superdisintegrants in pharmaceutical tablets.

#### 4.1.2. Swelling Capacity and Solubility Characteristics

The swelling power and solubility characteristics of NSS and SSG are presented in the table. SSG exhibits a greater swelling capacity compared to NSS, primarily due to the introduction of sodium salt or carboxymethyl groups, which disrupt and weaken the hydrogen bonds within the starch granules. This structural disruption facilitates water penetration into the granules, thereby enhancing their ability to swell. Furthermore, the weakening of hydrogen bonds following carboxymethylation promotes the release of amylose chains, which contributes to the increased solubility of the modified starch.

#### 4.1.3. Differential Scanning Calorimetry (DSC)

Differential scanning calorimetry (DSC) is a method used to identify the heat flow related to order–disorder transitions and provides a numerical assessment of gelatinization. The gelatinization endotherm determined through DSC offers a comprehensive evaluation of the gradual breakdown of the long-, medium-, and short-range order within starch granules when subjected to excessive water heating [26].

**Figure 3:**
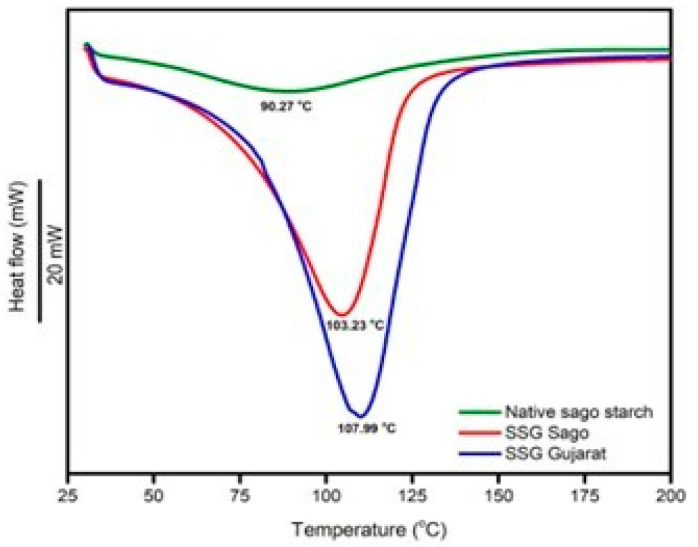
DSC thermogram of native sago starch, optimized SSG sago, and SSG Gujarat.

The thermal properties of materials can be analyzed using differential scanning calorimetry (DSC). In Figure 3, the DSC curves of native sago starch, SSG sago, and SSG Gujarat are presented. The gelatinization temperature of native sago starch is 90.27 °C, while it increases to 103.23 °C for SSG sago. This increase in the gelatinization temperature can be attributed to improved crystal perfection, resulting in a more thermally stable starch. The gelatinization temperature of SSG sago is similar to that of the commercial SSG Gujarat, which is 107.99 °C. The endothermic peak observed in the DSC curves is caused by the gelatinization temperature of sago starch. Similar observations have been reported by Ahmad et al. [27]. Native starch contains large amylopectin molecules, which are part of crystallites in granular layers. The non-crystalline or amorphous phase of these molecules connects the crystalline layers, forming granular birefringence. When heated with water, the penetration and swelling of the amorphous regions are limited due to the presence of crystallites. This limits the movement of starch chains and restricts water penetration and the swelling process. At high temperatures, the unstable crystallites undergo disruption or melting, resulting in the loss of birefringence. Therefore, DSC records the heat uptake (endothermic transition) of the sample [26].

#### 4.1.4. FT-IR Spectrometer Analysis

**Figure 4:**
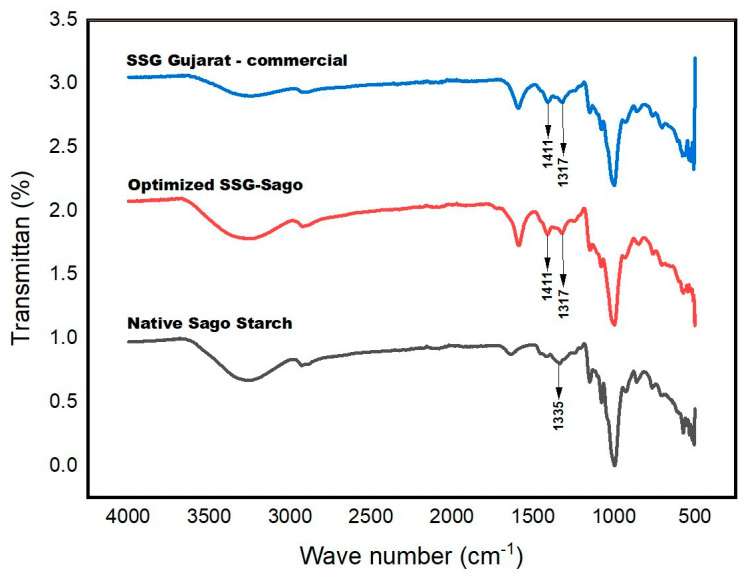
The FTIR spectra of native sago starch, optimized SSG sago, and SSG Gujarat.

The Fourier Transform Infrared (FTIR) spectrum of sago starch displays characteristic absorption bands associated with the starch backbone, as shown in Figure 4. A prominent absorption band at 3293 cm^−1^ corresponds to the O–H stretching vibration. While O–H stretching typically appears in the 3700–3500 cm^−1^ range, the peak observed in sago starch is shifted toward a lower wavenumber. This shift is likely due to the weakening of O–H bonds as a result of intermolecular hydrogen bonding within the glycosidic rings, causing the absorption to fall within the 3400–3200 cm^−1^ range.

Additionally, an absorption band at 2929 cm^−1^ is attributed to C–H stretching vibrations. A weak band at 1627 cm^−1^ may indicate the presence of tightly bound water molecules in the starch matrix. The symmetric stretching of –CH_2_ is observed at 1333 cm^−1^. Within the glycosidic structure, the C–O stretching vibrations of the C–O–C and C–O–H groups are represented by broad bands in the 1100–990 cm^−1^ range. Furthermore, the weak and broad bands observed between 930–600 cm^−1^ are likely associated with out-of-plane O–H bending and C–H deformation vibrations [27,28].

The FTIR spectra of the SSG samples display the typical absorption bands associated with the starch backbone, along with several additional peaks. Notably, new absorption bands appear at 1587 cm^−1^ and 1416 cm^−1^, indicating the presence of –COO^−^Na^+^groups resulting from substitution on the starch molecular chains. The C=O stretching frequency is observed to be lower than that of the original carboxylic acid, which can be attributed to resonance effects. Upon ionization, the formation of the carboxylate (COO^−^) group leads to resonance delocalization between the two C–O bonds. Consequently, the original carbonyl absorption band disappears and is replaced by two distinct bands—one in the 1610–1550 cm^−1^ range and the other between 1400–1300 cm^−1^—corresponding to the asymmetric and symmetric stretching vibrations of the COO− group.

An absorption band corresponding to C–O stretching was observed at 1004 cm^−^¹. Additionally, a peak at 1321 cm^−1^ may result from overlapping in-plane bending vibrations of –CH_2_ and O–H groups. A noticeable shift in the O–H stretching band was also recorded, with the peak moving to 3339 cm^−1^ and a decrease in its intensity. This shift and reduction in intensity are likely due to interactions between hydroxyl groups and the introduced carboxyl groups. The diminished O–H band may also reflect the partial substitution of hydroxyl groups by –CH_2_COONa during the etherification process. Similar observations regarding the changes in O–H vibration were reported by Fang et al. [29] during the modification of potato starches.

#### 4.1.5. Pasting Properties

The pasting profile describes the changes in starch viscosity as it undergoes thermal treatment, encompassing both heating and cooling stages. Table 6 presents a comparative analysis of the pasting behavior observed in the NSS, CRL-S, and SSG samples.

#### 4.1.6. Surface Morphology Analysis via SEM

**Figure 5:**
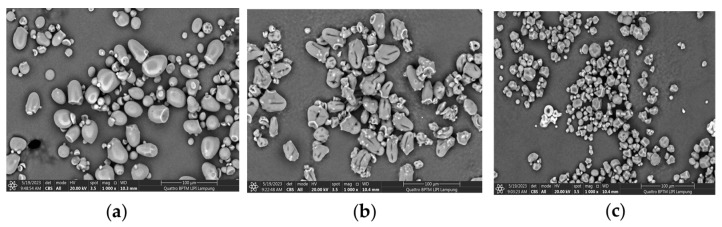
Morphology comparison of NSS (**a**); optimized SSG sago starch (**b**); and SSG Gujarat, a commercial potato starch (**c**).

The environmental Scanning Electron Microscope (Quattro S, Thermo Fisher Scientific, USA) was used to analyze the morphology of native sago starch (NSS) and sodium starch glycolate (SSG). Figure 5 displays micrographs of both NSS and SSG. The NSS grains exhibited smooth surfaces and a relatively uniform elliptical shape, with similar sizes. In contrast, the SSG granules had overhanging surfaces, and some areas on the granular surface showed shrinkage and cracking. Carboxymethylation caused changes in the shape of the granules due to the alkaline conditions. Strong alkaline conditions had a significant impact on the starch granules, leading to the loss of their crystal structure. Carboxymethylation likely occurred both on the surface and inside the starch granules. These findings provide evidence of starch modification, which aligns with previous research.

#### 4.1.7. X-Ray Diffraction Analysis—Crystallinity

**Figure 6:**
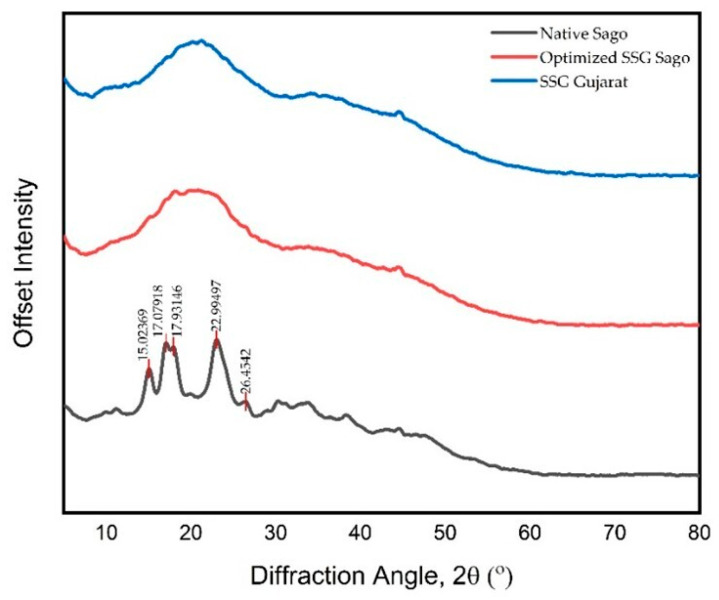
XRD diffractogram of native sago starch, optimized SSG sago, and SSG Gujarat.

X-ray diffraction (XRD) is a reliable technique for assessing the degree of crystallinity in polymers. In starch, which exhibits semi-crystalline characteristics, crystallinity is primarily influenced by the amylopectin content. The XRD pattern of native granular sago starch, shown in Figure 6, displays sharp peaks corresponding to crystalline regions, while the diffuse background indicates the amorphous phase. The broad nature of the peaks suggests the presence of small crystallites within the starch granules, consistent with XRD principles that associate broadened diffraction peaks with imperfect or nano-sized crystals.

The diffraction profile of sago starch reveals a small peak at 15.68°, twin peaks at 17.1° and 17.9°, and additional peaks at 23.0° and 26.0°. These findings are consistent with previous studies by Yanuar et al. and Ahmad et al. [27,28,29,30], who reported similar diffraction patterns for sago starch and proposed that its crystalline structure is intermediate between that of cereal and potato starches.

In contrast, the XRD pattern of SSG shows a complete loss of the crystalline structure observed in native starch. The characteristic peaks of unmodified starch are absent, indicating that the material has been fully converted into an amorphous form. The transformation of SSG into an amorphous structure, as indicated by the absence of characteristic crystalline peaks in the XRD pattern, plays a significant role in enhancing its performance as a superdisintegrant. Amorphous materials generally have a higher free volume and greater molecular mobility, which allows water molecules to penetrate more rapidly into the matrix. This increased water uptake promotes faster swelling and particle expansion, which are critical for disintegration. In contrast to crystalline regions that resist hydration due to tightly packed molecular arrangements, the amorphous structure facilitates immediate interaction with water, thus accelerating the breakdown of the tablet matrix. Therefore, the loss of crystallinity in SSG contributes directly to its improved disintegration efficiency in pharmaceutical formulations. This structural transformation leads to significantly enhanced solubility [31]. During the carboxymethylation process, starch molecules are exposed to an alkaline environment. The swelling of starch granules exerts mechanical stress on the surrounding crystalline regions, resulting in their deformation. As swelling progresses, the double-helical regions begin to unwind or separate, ultimately leading to the disruption of the crystalline structure [29].

In Table 7 above it can be explained that SSG (sodium starch glycolate) derived from sago presents several advantages over its potato-based counterparts (types A, B, and C), which are commonly used in pharmaceutical formulations. First, SSG sago has a significantly lower sodium content, ranging from 0.9% to 2.3%, compared to the 7.0% found in types A and B and the 1.0% in type C, making it more suitable for applications requiring strict sodium control. Additionally, the pH of SSG sago is closer to neutral (6.8–7.8), offering improved compatibility for formulations where a more neutral or slightly alkaline environment is preferred, compared to the potato-based types which exhibit a pH range of 5.5–7.5. SSG sago also demonstrates a lower loss on drying (1.7%), indicating a superior moisture stability and potentially enhancing the product shelf life and consistency. The assay for sodium in SSG sago is within the range of 2.7–4.3%, comparable to potato-based types; however, its lower sodium content offers added flexibility in controlling the overall sodium levels in formulations. Furthermore, SSG sago requires more precise identification methods, including IR absorption spectrum analyses and uranyl acetate tests, which provide higher accuracy in quality control compared to traditional iodine-based colorimetric tests. Lastly, sourcing SSG from sago starch rather than from potatoes could offer significant logistical and economic advantages, particularly in regions where sago is more abundant and cost-effective. These characteristics suggest that SSG sago is a promising alternative to potato-based SSG, particularly in applications that require improved stability, sodium control, and pH compatibility.

#### 4.1.8. Test Results for Mefenamic Acid FDT Physical Characteristics

##### Organoleptic Test Results

The organoleptic evaluation of mefenamic acid FDTs focuses on assessing the tablets’ physical characteristics, such as shape, color, surface texture, and overall appearance. Below are the findings from the visual organoleptic tests conducted on the mefenamic acid FDTs which are described in detail in Table 8:

##### Weight Uniformity Test Results

**Figure 7:**
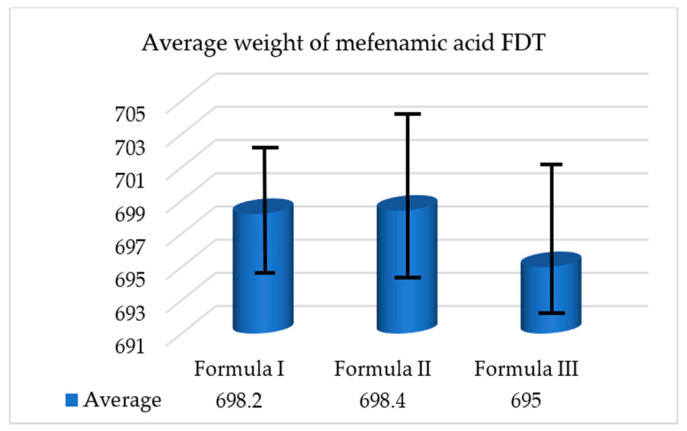
Average weight of mefenamic acid FDTs.

The purpose of weight uniformity testing for mefenamic acid FDTs is to assess the consistency in weight among the tablets that were manufactured. This evaluation involves collecting 20 FDT samples from each formulation, measuring their individual weights, and subsequently determining the average weight of the tablets. The test procedure was repeated thrice for every formulation to ensure accuracy. In Figure 7 below is presented the average weights of mefenamic acid FDTs from each respective formula.

The USP mandates certain criteria for ensuring the uniformity of tablet weights. According to these requirements, if tablets are individually weighed, the weight deviation of no more than two tablets should exceed the average weight deviation specified in column A. Additionally, there should be no single tablet whose weight deviates more than the average weight deviation mentioned in column B. In the case of mefenamic acid FDTs, which have an average weight of 700 mg, the allowable average weight deviation is 5% for column A and 10% for column B. The results indicate that the mefenamic acid FDTs meet the tablet weight uniformity standards set by the USP [32].

##### Tablet Hardness Test Results

The purpose of conducting hardness testing on the mefenamic acid FDTs is to assess the tablets’ resistance to pressure encountered during packaging, distribution, and transportation to end-users. The test involved measuring the hardness of 10 FDT samples of mefenamic acid from each formula using a hardness tester (PharmaTest, Hainburg, Germany) [33]. Each formula underwent three repetitions of the test. The results of the tablet hardness values are as follows:

The results displayed in Table 9 indicate the outcomes of the variance tests conducted on each formula. Formula I exhibited the highest level of hardness when compared to Formulas II and III, suggesting that the inclusion of the SSG content does impact the hardness of the FDTs. Typically, a well-made tablet is expected to possess a hardness ranging from 4 to 8 kg [33]. The findings reveal that the tablets in Formulas I, II, and III all satisfy the tablet hardness criteria outlined by the USP.

##### Tablet Friability Test Results

The purpose of the mefenamic acid FDT fragility test is to assess the overall fragility of the FDTs. This test helps determine whether the tablets can withstand shocks during packaging, shipping, and consumer handling without breaking. Prior to conducting the test, the mefenamic acid FDT samples were thoroughly cleaned using a brush. Then, 13 mefenamic acid FDT samples, each weighing an average of 700 mg per tablet, were weighed. The samples were placed in a friabilitor and rotated at a speed of 25 + 1 rpm for 100 rotations [33]. Afterward, the FDTs were cleaned again with a brush to remove any adhering powder, and the total weight of each tablet was measured once more. The following results explained in Table 10 represent the fragility test outcomes for FDTs of mefenamic acid.

Based on the friability test results, it is evident that the mefenamic acid FDTs failed to meet the tablet friability requirements as they exceeded the allowable weight loss of 1%. The data table displays the varying fragility values for each formula, with Formula III exhibiting a higher fragility value (1.75 ± 0.085%) compared to Formula II (1.54 ± 0.086%) and Formula I (1.32 ± 0.079%). This discrepancy in the fragility percentages between the formulas with and without the 5% SSG excipient indicates that the addition of SSG levels influences the fragility of FDTs.

##### Disintegration Time Test Results

The disintegration time testing of the mefenamic acid FDTs aimed to determine the time needed for complete disintegration. Each tablet was placed in a basket within an Erweka disintegration apparatus (PharmaTest, Germany) filled with distilled water at 37 ± 0.5 °C [33]. Subsequently, the apparatus was activated, and the disintegration time for each mefenamic acid FDT formula was noted. In Table 11 below are the outcomes of the disintegration time testing for the mefenamic acid FDTs utilizing sodium starch glycolate as a superdisintegrant.

As per the *European Pharmacopoeia* guidelines, orodispersible tablets, also known as FDTs, are required to disperse or disintegrate within three minutes. The table provided displays the test results for each formula. Formula I exhibited the longest disintegration time compared to Formula II and Formula III (refer to the table). This is because Formula I served as a control formula without any disintegrants added. On the other hand, Formula III demonstrated the quickest disintegration time among the mefenamic acid FDTs, with an average time of 1.14 ± 0.036. This indicates that the increasing levels of SSG have an impact on the disintegration time of FDTs. The results of the formula testing indicate that mefenamic acid FDTs with the inclusion of SSG sago meet the requirements of the USP and exhibit a superior disintegration time compared to tablets incorporating the existing SSG available on the market. Although the disintegration results demonstrate the effectiveness of SSG sago under standard USP conditions using distilled water, further evaluation using biorelevant media, such as simulated gastric fluid (SGF) and simulated intestinal fluid (SIF), is recommended. These additional studies would provide a more comprehensive assessment of the disintegration behavior and functional performance of the superdisintegrant under physiological conditions, enhancing its relevance for real-world pharmaceutical applications.

## 5. Conclusions

This study successfully optimized the production of sago-starch-derived sodium starch glycolate (SSG) using a Response Surface Methodology (RSM) and demonstrated its potential as an effective superdisintegrant in pharmaceutical formulations. The optimized SSG product derived from sago starch exhibited a superior performance in mefenamic acid tablet formulation, meeting the requirements of the USP and providing a sustainable and economically viable SSG source. The findings revealed that the optimal parameters were a reaction temperature range of 45–55 °C, an SMCA ratio of 0.75–1.5, and a reaction time of 120–240 min, with the maximum predicted DS value of 0.24 being achieved at a temperature of 55 °C, an SMCA ratio of 1.185, and a reaction time of 125 min. The resulting formulation, validated under optimum process conditions, had a mean validated DS value of 0.246 ± 0.021, indicating the accuracy of the simulated model. This study contributes to the development of more effective drug delivery systems and promotes sustainable agriculture in Indonesia by utilizing sago starch as a potential source for SSG production. The findings of this study can be further explored to unlock numerous benefits, such as increasing the added value of sago starch, creating potential new economic opportunities in pharmaceutical industries, and promoting the sustainable development of Indonesian agriculture.

## Figures and Tables

**Table 1:** Statistical summary of RSM.

Source	Standard Deviation	Lack-of-Fit	R^2^	Adjusted R^2^	Predicted R^2^	PRESS	*p*-Value	Remarks
Linear	0.0335	0.0037	0.7872	0.7473	0.6159	0.0324	<0.0001	
2Fl	0.0329	0.0034	0.8334	0.7565	0.6570	0.0289	0.3476	
Quadratic	0.0127	0.2140	0.9809	0.9637	0.8864	0.0096	<0.0001	Suggested
Cubic	0.0095	0.6004	0.9935	0.9794	0.9071	0.0078	0.1172	Aliased

**Table 2:** Data showing the degree of substitution responses for SSG.

Run	A	B	C	Response (R) of DS	
	Reaction Temperature (°C)	SMCA Ratio	Reaction Time (min)	Experimented	Predicted
1	45	0.75	120	0.122 ± 0.001	0.1215
2	55	0.75	120	0.211 ± 0.001	0.1896
3	45	1.5	120	0.141 ± 0.001	0.1348
4	55	1.5	120	0.267 ± 0.002	0.2661
5	45	0.75	240	0.133 ± 0.001	0.1245
6	55	0.75	240	0.215 ± 0.002	0.2106
7	45	1.5	240	0.185 ± 0.001	0.1966
8	55	1.5	240	0.356 ± 0.001	0.3460
9	41.59	1.125	180	0.057 ± 0.001	0.0471
10	58.41	1.125	180	0.224 ± 0.001	0.2299
11	50	0.494328	180	0.142 ± 0.001	0.1485
12	50	1.75567	180	0.284 ± 0.001	0.2735
13	50	1.125	79.09	0.205 ± 0.001	0.2127
14	50	1.125	280.91	0.294 ± 0.001	0.2824
15	50	1.125	180	0.195 ± 0.001	0.2038
16	50	1.125	180	0.210 ± 0.001	0.2038
17	50	1.125	180	0.206 ± 0.001	0.2038
18	50	1.125	180	0.201 ± 0.001	0.2038
19	50	1.125	180	0.209 ± 0.001	0.2038
20	50	1.125	180	0.225 ± 0.001	0.2038

**Table 3:** The ANOVA of the degree of substitution for the SSG response surface quadratic model.

Source	Sum of Squares	DOF *	Mean Square	F-Value	*p*-Value	
Model	0.0826	9	0.0092	57.09	<0.0001	Significant
A	0.0410	1	0.0410	254.97	<0.0001	
B	0.0189	1	0.0189	117.30	<0.0001	
C	0.0064	1	0.0064	40.02	<0.0001	
AB	0.0020	1	0.0020	12.43	0.0055	
AC	0.0002	1	0.0002	1.05	0.3292	Not significant
BC	0.0017	1	0.0017	10.74	0.0083	
A^2^	0.0077	1	0.0077	47.77	<0.0001	
B^2^	0.0001	1	0.0001	0.5745	0.4660	Not significant
C^2^	0.0034	1	0.0034	21.43	0.0009	
Residual	0.0016	10	0.0002			
Lack-of-Fit	0.0011	5	0.0002	2.12	0.2140	Not significant
Pure Error	0.0005	5	0.0001			
Correction Total	0.0843	19				
R^2^	0.9809					
Adjusted R^2^	0.9637					
Predicted R^2^	0.8864					
Adequate Precision	33.5819					
C.V%	6.22					

* DOF = degree of freedom.

**Table 4:** Functional characterization of amylose content, DS, and NaCl measurement.

Run	Response (R) of Na levels, DS and NaCl		
	Na Levels	DS	NaCl
1	8.16 ± 0.001	0.122 ± 0.001	0.082 ± 0.001
2	14.15 ± 0.001	0.211 ± 0.001	0.248 ± 0.001
3	9.34 ± 0.001	0.141 ± 0.001	0.041 ± 0.001
4	16.77 ± 0.002	0.267 ± 0.002	1.242 ± 0.001
5	8.86 ± 0.001	0.133 ± 0.001	0.124 ± 0.001
6	13.77 ± 0.002	0.215 ± 0.002	0.248 ± 0.002
7	12.04 ± 0.002	0.185 ± 0.001	0.248 ± 0.001
8	21.47 ± 0.002	0.356 ± 0.001	0.621 ± 0.002
9	3.93 ± 0.001	0.057 ± 0.001	0.248 ± 0.001
10	14.31 ± 0.002	0.224 ± 0.001	0.497 ± 0.001
11	9.40 ± 0.001	0.142 ± 0.001	0.124 ± 0.001
12	17.68 ± 0.001	0.284 ± 0.001	1.988 ± 0.002
13	13.20 ± 0.002	0.205 ± 0.001	1.739 ± 0.002
14	18.23 ± 0.004	0.294 ± 0.001	0.497 ± 0.001
15	12.60 ± 0.002	0.195 ± 0.001	0.124 ± 0.001
16	13.53 ± 0.002	0.210 ± 0.001	0.124 ± 0.001
17	13.29 ± 0.001	0.206 ± 0.001	2.174 ± 0.001
18	12.98 ± 0.001	0.201 ± 0.001	0.248 ± 0.001
19	13.42 ± 0.002	0.209 ± 0.001	2.236 ± 0.001
20	14.39 ± 0.002	0.225 ± 0.001	0.372 ± 0.001
SSG Gujarat	19.69 ± 0.002	0.322 ± 0.001	3.852 ± 0.002

**Table 5:** Effect of crosslinking and carboxymethylation on the functional characteristics of sago.

Characteristics	NSS	CRL-SS	SSG-S	SSG-GJ
Amylose Content (%)	20.70 ± 0.08 ^a^	6.10 ± 0.12 ^b^	4.10 ± 0.08 ^a^	3.898 ± 0.08 ^a^
Swelling Power (g/g)	16.50 ± 0.27 ^b^	3.73 ± 0.12 ^b^	25.49 ± 0.09 ^b^	28.05 ± 0.14 ^b^
Solubility (%)	29.36 ± 0.09 ^b^	0.053 ± 0.08 ^a^	0.635 ± 0.07 ^b^	0.889 ± 0.08 ^a^
Moisture Content (%)	16.10 ± 0.04 ^b^	10.21 ± 0.07 ^b^	8.49 ± 0.14 ^b^	7.08 ± 0.04 ^b^
pH	6.42 ± 0.04 ^b^	7.11 ± 0.12 ^b^	7.035 ± 0.09 ^b^	7.16 ± 0.14 ^b^

^a,b^ Mean values within the same column that are followed by different letters indicate a statistically significant difference.

**Table 6:** Pasting properties of native sago starch, optimized SSG sago, and SSG Gujarat.

Parameter	Sago Starch			
	NSS	CRL-S	SSG-S	SSG Gujarat
Pasting Temperature (°C)	71.10 ± 1.00 ^a^	30.1 ± 1.00 ^a^	30.4 ± 1.00 ^a^	30.1 ± 1.00 ^a^
Peak Viscosity (BU)	585.67 ± 12.90 ^b^	4.67 ± 2.90 ^b^	0	0
Final Viscosity (BU)	432.33 ± 12.34 ^b^	2.32 ± 2.34 ^b^	0	0
Breakdown Viscosity (BU)	321.5 ± 26.63 ^a^	2.15 ± 6.63 ^a^	0	0
Setback Viscosity (BU)	175.67 ± 5.77 ^b^	−1.67 ± 6.77 ^b^	616.47 ± 5.77 ^b^	601.37 ± 4.77 ^b^

^a,b^ Mean values within the same column that are followed by different letters indicate statistically significant differences.

**Table 7:** Pharmacopeia specifications for various brands of sodium starch glycolate and optimized sodium starch glycolate.

Tests	Type A	Type B	Type C	SSG Sago
**Definition**	Sodium salt of crosslinked partly O-carboxymethylated potato starch.	Sodium salt of crosslinked partly O-carboxymethylated potato starch.	Sodium salt of a crosslinked by physical dehydration partly O-carboxymethylated starch.	Sodium salt of crosslinked partly O-carboxymethylated sago starch.
**Na**	2.8–4.2%	2.0–3.4%	2.8–5.0%	2.7–4.3%
**pH**	5.5–7.5	3.0–5.0	5.5–7.5	6.8–7.5
**LOD**	≤10.0%	≤10.0%	≤7.0%	1.794
**Sodium chloride**	7.0%	7.0%	1.0%	0.04–2.23%
**Sodium glycolate**	2.0%	2.0%	2.0%	NA
**Assay (of Na)**	2.8–4.2%	2.0–3.4%	2.8–5.0%	2.7–4.3%
**Size**	30–100 µm	30–100 µm	30–100 µm	30–100 µm
**Identification**	The IR absorption spectrum, as per reference spectrum.	Iodine—blue color.	(1) K-antimonate-White ppt. (2) Mg-Uranyl acetate-yellow ppt.	The IR absorption spectrum. as per reference spectrum. 1411 sodium salt.

**Table 8:** The results of the organoleptic test for mefenamic acid FDTs.

Organoleptic	Formula I	Formula II	Formula III
Shape	The shape is characterized by its round form with flat surfaces on both the top and the bottom.	The shape is characterized by its round form with flat surfaces on both the top and the bottom.	The shape is characterized by its round form with flat surfaces on both the top and the bottom.
Color	Dark white.	Dark white.	Dark white.
Surface texture	The surface is smooth and without defects.	The surface is smooth and without defects.	The surface is smooth and without defects.
Appearance	Homogeneous, no spots or stains.	Homogeneous, no spots or stains.	Homogeneous, no spots or stains.

Note: Formula I represents a formula that lacks SSG (control), while Formula II incorporates SSG sago as a superdisintegrant. On the other hand, Formula III utilizes SSG Gujarat, a commercial potato starch, as a superdisintegrant. It is evident from the provided table that the mefenamic acid FDT formulas exhibit a consistent shape, color, texture, and physical appearance.

**Table 9:** FDT hardness value of mefenamic acid.

Formula	Mean ± SD (kg)
I	7.02 ± 1.08
II	5.64 ± 0.83
III	5.48 ± 0.396

**Table 10:** Results of mefenamic acid FDT friability test.

STD	No	Initial Weight	Weight After	Percent Friability (%)	Mean ± SD (%)
Formula I	1	7.03	6.94	1.28	1.32 ± 0.079
	2	7.04	6.95	1.27	
	3	7.06	6.96	1.41	
Formula II	1	6.91	6.80	1.59	1.54 ± 0.086
	2	6.92	6.81	1.58	
	3	6.94	6.84	1.44	
Formula III	1	7.02	6.90	1.71	1.75 ± 0.085
	2	7.01	6.88	1.85	
	3	7.03	6.91	1.70	

**Table 11:** Disintegration time testing for mefenamic acid FDTs.

STD	Disintegration Time (min)						Mean ± SD (min)
Formula I	> 0	>30	>30	>30	>30	>30	>30 ± 0.01
Formula II	1.09	1.12	1.16	1.10	1.17	1.17	1.14 ± 0.036
Formula III	1.32	1.36	1.49	2.00	2.10	2.24	1.75 ± 0.40

## Data Availability

Suggested Data Availability Statements are available in section “MDPI Research Data Policies” at https://www.mdpi.com/ethics.
